# A Dataset of Plausible Proton Transfer Steps for Arrow-Pushing Mechanisms

**DOI:** 10.1038/s41597-025-06490-8

**Published:** 2026-01-10

**Authors:** Alexander E. Dashuta, Ryan J. Miller, Pierre Baldi, Thomas Sander, David L. Van Vranken

**Affiliations:** 1 Department of Chemistry, University of California, Irvine, CA 92697 USA; 2 Department of Computer Science, University of California, Irvine, CA 92697 USA; 3 Alipheron AG, Schlossweg 63, 4143 Dornach, Switzerland

**Keywords:** Cheminformatics, Reaction mechanisms

## Abstract

Proton transfers are fundamental steps in polar reaction mechanisms. We generated a large dataset of over 51 million kinetically plausible proton transfer steps between heteroatoms from about 8,000 acids and conjugate bases with experimental aqueous p*K*_a_s, spanning p*K*_a_ values from −15 to +37. Rate factors were estimated at 25 °C using a simplified Eigen equation with p*K*_a_s but without statistical factors. Steps with estimated rate constants ≥ 10^3^ M^−1^ s^−1^ were included in the final dataset. Additionally, 5,043 proton transfer steps from carbon acids to heteroatom bases were estimated using the Eigen-Bernasconi equation based on reported intrinsic rate constants and Brønsted β values. Carbon proton transfers with rate constants ≥ 10^3^ M^−1^ s^−1^ were added to the final dataset. Each entry was encoded in SMIRKS format with electron-flow specification for machine learning compatibility. Diversity of structure was prioritized over diversity of conditions; calculated rate constants are expected to be accurate in aqueous environments. This approach and dataset should prove valuable for training models to predict stepwise mechanistic pathways.

## Background & Summary

Proton transfer steps are particularly important in stepwise mechanistic pathways for organic transformations in synthesis, biochemical processes, and environmental chemistry. For example, an intermediate organic chemistry text^1^ has over 70% (900 out of 1,269) diverse polar transformations that involve stepwise mechanisms with at least one proton transfer step. There are many datasets of acidic species with equilibrium p*K*_a_ values^2–5^, but no large datasets of solution phase acid-base proton transfers suitable for training and learning (Table 1). For example, the Notre Dame Radiation Laboratory (NDRL) / NIST Solution Kinetics Database (through 1995) has abundant rate data, focusing on radicals^6^. Few of the 23,675 records involve proton transfers: none of the 11 records for pyridine involve proton transfer steps, and only 1 out of 26 records for acetic acid involve proton transfers. The proprietary Reaxys dataset has a large number of rate constants, but few for proton transfers^7^.

**Table 1:** Datasets of Proton Transfers with Rate Information.

Dataset Source	Proton Transfers	Entries with Rate Data	Availability	Ref
NDRL / NIST Solution Kinetics Database	Radical species with few proton transfers	NA	Public	^6^
Reaxys	2,741 Reaction Type contains “proton” or is “acid-base reaction”	21 proton transfers with subject “Rate Constant”	Proprietary	^7^
Plausible Proton Transfer Steps (this work)	51,510,157	24,890,236 with calculated or experimental log *k*_1_	Public	

Datasets have a significant advantage over general equations because exceptional examples can easily be added to a dataset, but not to an equation^8^. The most common datasets focus on transformations, without revealing the underlying stepwise mechanistic pathways. More recently, efforts have focused on databases of individual mechanistic steps^9^. The large NIST Chemical Kinetics database lists elementary reaction steps along with composite processes, mostly for gas-phase reactions^10^. Frenklach and workers created a dataset of mechanistic steps, with rate factors, from combustion processes^11,12^. Green, West, and coworkers have created a much larger dataset of mechanistic steps with rate constants for combustion processes^13^. Coley and coworkers created a large dataset of mechanistic steps and used it to train for prediction of stepwise pathways^14^. Grzybowski and coworkers generated a large dataset of carbenium ion rearrangements, with energetic parameters for prediction of rearrangement pathways^15^. Jung, Han, and coworkers also reported a large dataset of mechanistic steps to support prediction of polar reaction pathways^16^. We set out to create a large dataset of plausible proton transfer steps, each with electron-flow specification corresponding to curved arrows^17^. The term “plausible” (as opposed to “proven”) accounts for wide variations in factors such as species concentrations and solvation. Our goal was to use equilibrium aqueous p*K*_a_s to estimate rate constants for proton transfer steps^18,19^, and include in the dataset those proton transfer steps expected to be fast at room temperature using conservative boundaries.

**Fig. 1:**
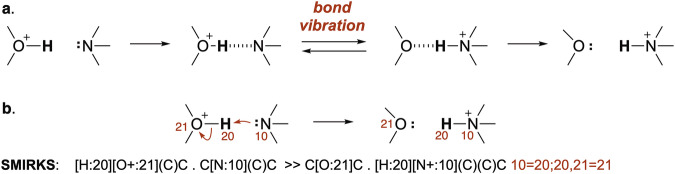
Proton transfers have been represented as a stepwise or concerted process. (**a**) The 3-step Eigen mechanism for proton transfers. (**b**) The common 1-step arrow-pushing depiction of proton transfers, with SMIRKS code.

Simple, one-step representations of proton transfers belie a more complex process. The Eigen mechanism^20,21^ for proton transfer involves three steps (Fig. 1a): i) formation of a hydrogen bond between acid and base, ii) transfer of proton from acid to base through a bond vibration, and iii) dissociation of the conjugate acid and the conjugate base. Following arrow-pushing convention, we treat proton transfers as a *one-step process* with no hydrogen-bonded intermediates (Fig. 1b). The exclusion of hydrogen-bonded intermediates is particularly important for consistency with Lewis representations in SMILES and SMIRKS text formats, which are commonly used for machine learning^17,22–25^.

## Methods

### Proton Transfers to and from Heteroatoms

**Fig. 2:**
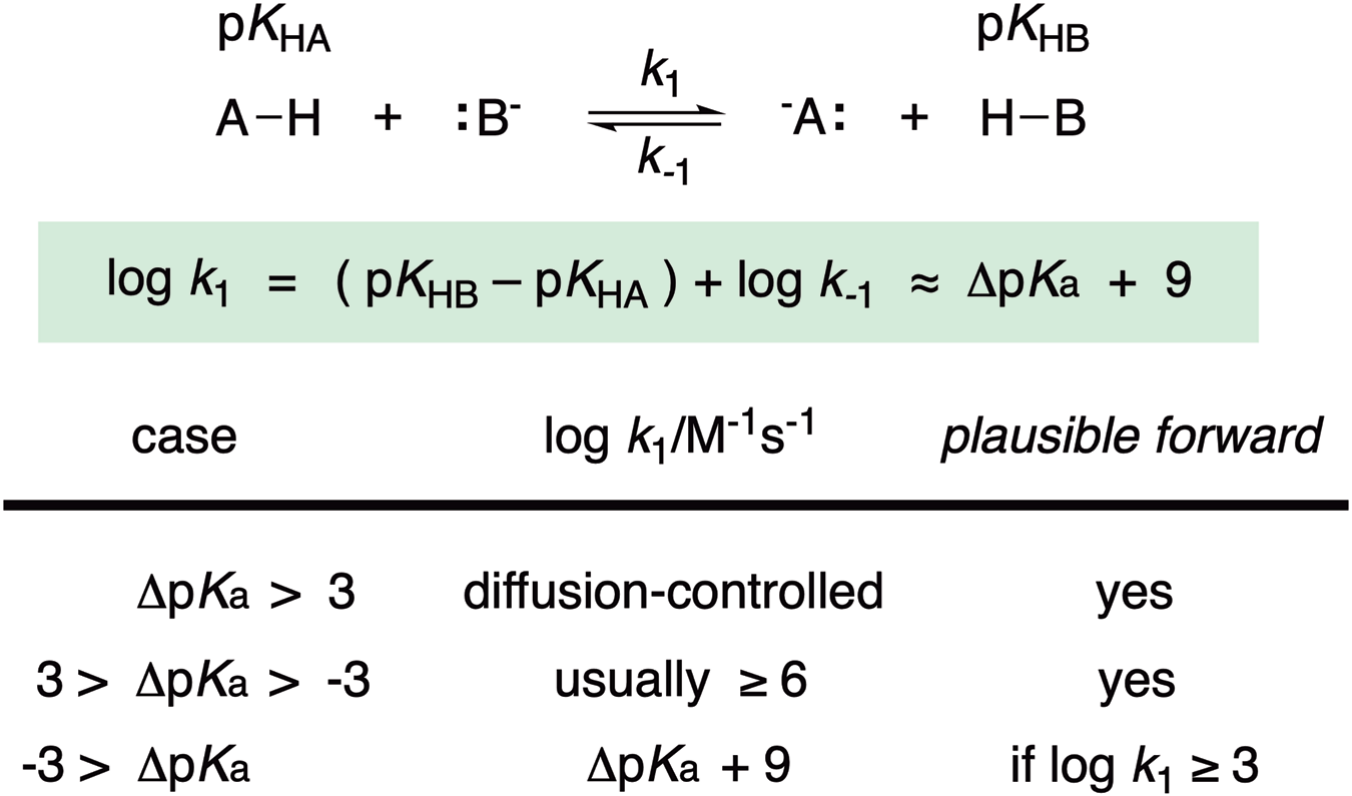
Relationship between equilibrium and rate constants for proton transfers involving heteroatoms.

For proton transfers to and from heteroatoms, the second-order rate constants can often be predicted when the p*K*_HA_ of the acid and the p*K*_HB_ of the base are known^26^. Under equilibrium (or pre-equilibrium) conditions, when the acid is at least 1000 times more acidic than the conjugate acid (p*K*_HB_ − p*K*_HA_ ≥ 3, Fig. 2), the forward proton transfer is diffusion-controlled (*k*_1_ = 10^9^ M^−1^ s^−1^). These diffusion-controlled proton transfer steps with rate factors > 10^9^ M^−1^ s^−1^ should be considered plausible and are included in the dataset with log *k*_1_ = 9. When the backward proton transfer is diffusion-controlled (−3 > ∆p*K*_a_), the rate constant is readily estimated from the simplified relationship (Eq. 1), which ignores minor statistical factors for the number of acidic sites on the conjugate acid HB and the number of basic sites on the conjugate base^21^.

1 $$\log \,{k}_{1}=({\rm{p}}{K}_{{\rm{H}}{\rm{B}}}-{\rm{p}}{K}_{{\rm{H}}{\rm{A}}})+\log \,{k}_{-1}\approx {\rm{\bigtriangleup }}{\rm{p}}{K}_{{\rm{a}}}+9$$

The mathematical relationship between rate and equilibrium doesn’t hold for proton transfers between heteroatoms with comparable acidity (3 > ∆p*K*_a_ ≥ −3). The range over which ∆p*K*_a_ ceases to correlate with the rate constant depends on the heteroatoms^27^, while the range is larger for thiols and carbon acids; carbon acids are dealt with separately below^28^. Even though the relationship given by Eq. (1) is unreliable for proton transfers between species of comparable acidity, proton transfers involving O, N, and S acids with comparable acidity are typically very fast in each direction (*k*_1 _≥ 10^6^ M^−1^ s^−1^)^29,30^. We assume the same holds true for Se (selenols)^31^. Therefore, proton transfers between heteroatom species with comparable acidity are included in the dataset, but no log *k*_1_ is displayed.

In cases of thermodynamically *unfavorable* proton transfers where the product acid is more than 1000 times more acidic than the reactant acid (−3 > ∆p*K*_a_), the rate constants can still be calculated, but what rate constant is the cutoff for plausibility? Our goal for this dataset is to be conservative, excluding many plausible proton transfers in order to have greater confidence in the proton transfer steps in the dataset. In the standard state (25 °C), where both acid and base are present at 1 M, a second-order rate constant *k*_1_ = 10^−1^ M^−1^ s^−1^ corresponds to a half-life on the order of seconds (from t_1/2_ = 1 / (*k* • [HA]_0_), where [HA]_0_ = [HB]_0_), which seems quite plausible. However, the concentrations of acidic and basic species are almost never 1 M and the solvent and other species will greatly impact proton transfer rates. To be more conservative, we include only those proton transfers predicted to be plausible at 0.1 mM where *k*_1_ ≥ 10^3^ M^−1^ s^−1^ corresponds to t_1/2_ < 10 s. This would also correspond to acid-base proton transfers with log *k*_1_ ≥ 3 and ∆p*K*_a_ ≥ −6.

### Assembly of Arrow-Pushing Steps for Proton Transfers Between Heteroatoms

With these criteria in mind, we utilized the rich set of p*K*_HA_ values tabulated in the DataWarrior dataset made by Sander and coworkers^32^. The DataWarrior dataset contains 7,913 entries with SMILES representations, temperatures, and p*K*_HA_ values; 1,002 entries without temperatures were assumed to be at room temperature. About half of the structures, represented in the conjugate base form, were converted to SMILES for the conjugate acid form. Aromatic molecules, except for tropylium cation and a few others, are represented in non-Kekulized SMILES forms. Entries with temperatures near the range from room temperature (25 °C) to physiological temperature (37 °C) were included. Four examples with p*K*_HA_ values below 15 °C and 6 examples with p*K*_HA_ values above 40 °C were discarded, leaving 7,913 acids. The set was culled to remove 185 entries for which the site of protonation, tautomeric form, or other aspects of structure could not be confirmed, leading to a total set of 7,728 acids. Carbon acids were dealt with in a different way, so 209 carbon acids were removed to leave 7,519 heteroatom acids in the list. However, HCN has been shown to behave as a normal Eigen heteroatom acid^33^ and was added to the dataset with a p*K*_a_ of 9.0. For 41 acids with more than one p*K*_HA_ value, the entry with the p*K*_a_ value closest to 7.0 was used, leaving 7,479 entries. Water (H_2_O) and hydronium ion (H_3_O^+^) were notably absent from the DataWarrior dataset. Traditional p*K*_a_ values for H_2_O (p*K*_a_ 15.7) and H_3_O^+^ (p*K*_a_ −1.7) have recently been revised by log(55 M), to values of 14.0 and 0, respectively^34,35^. We added H_2_O and H_3_O^+^ to our list of acids, using the newer values. The final set of heteroatom acids and bases taken from DataWarrior contained 7,481 entries.

To better represent mechanistic intermediates for which no experimental p*K*_a_s are available, p*K*_a_ values for a range of other heteroatom acids were taken from the well-known Reich tables^36^, from Guthrie’s estimates of p*K*_a_s for mechanistic intermediates^37,38^, plus a set of sixteen distinctive heteroatom functional groups not represented in the DataWarrior set. This additional set of 132 additional acids was added to the DataWarrior set for a total of 7,613 in the overall Heteroatom set.

For each entry, the acidic proton in the SMILES string was mapped as atom 20 and the attached heteroatom was mapped as atom 21, for subsequent use in arrow-pushing. A complementary set of 7,613 conjugate bases was generated and the basic atom was mapped as atom 10 for use in arrow-pushing specification.

From the Heteroatom set of 7,613 heteroatom acids and 7,613 heteroatom bases, all possible combinations (7,613^2^ = 57,957,769) of acid-base reactions were generated. Combinations are represented in SMIRKS format along with electron-flow specification^17^ where the basic atom mapped as 10 deprotonates the acidic proton mapped as 20 and the attached heteroatom mapped as 21. Each entry included fields for p*K*_HA_, p*K*_HB_, and ∆p*K*_a_. Transformations with ∆p*K*_a_ ≥ −6.00 (89%) were deemed plausible and were retained in the final dataset of 51,505,065 proton transfer steps between heteroatoms in SMIRKS format, ready for use in applications such as deep learning of stepwise mechanisms. Doubly-charged species are common in the Heteroatom dataset (6.3% of acids have net charge >  + 1 and 6.6% of bases have net charge < −1) so it is important for users to be aware that steps that form dications or dianions are likely to be less plausible in non-aqueous solvents.

**Fig. 3:**
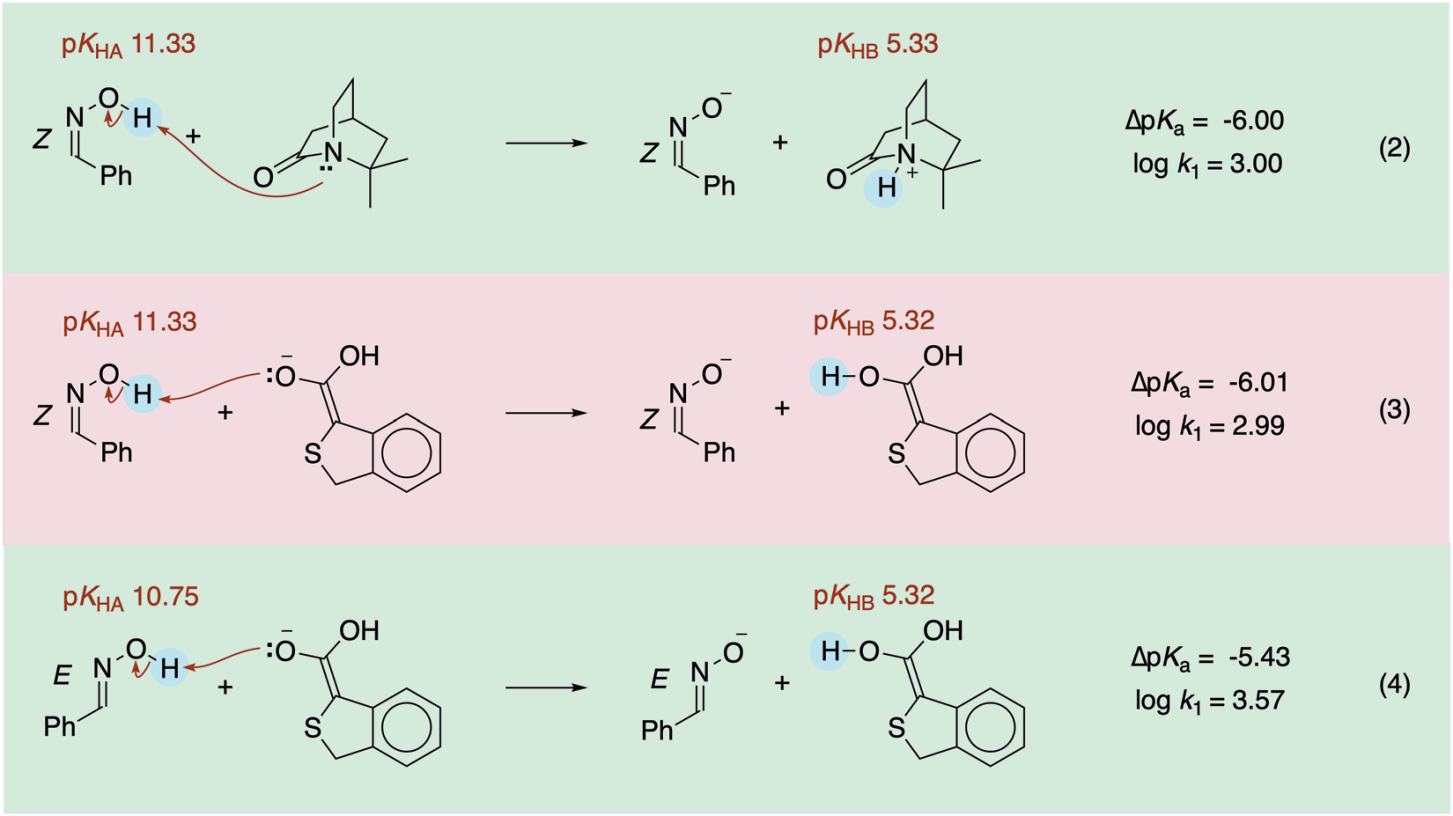
The precise cutoff of log *k*_1_ ≥ 3.00 leads to exclusion of some proton transfer steps yet each acid and base in Eq. 3 is still represented thousands of times in the dataset.

Using a precise cutoff of log *k*_1_ ≥ 3.00 may seem arbitrary because few of the tabulated p*K*_a_s were determined with ±0.01 accuracy. However, the cutoff removes only 6,452,704 (11%) out of the nearly 58 million proton transfer steps. The precise cutoff results, for example (Fig. 3), in inclusion of a proton transfer from a *Z* oxime to a quinuclidone (Eq. 2, log *k*_1_ = 3.00), but exclusion of a proton transfer from the same *Z* oxime to an enolate (Eq. 3, log *k*_1_ = 2.99). Interestingly, the same proton transfer involving the more acidic *E* oxime (p*K*_HA_ 10.75)^39^ is included in the dataset (Eq. 4, log *k*_1_ = 3.57). Fortunately, both the acid and the base in the excluded entry (Eq. 3) are well-represented in this combinatorial dataset with 4,473 and 7,242 occurrences of each, respectively.

### Proton Transfers Between Heteroatoms and Carbon

Rates of proton transfers to and from carbon are generally slower than corresponding rates for proton transfers between heteroatoms^27^. We set aside proton transfers between carbon atoms, with the expectation that carbon-to-carbon proton transfers will be much slower than proton transfers involving at least one heteroatom^40–43^. Rate constants for deprotonation of carbon acids by heteroatom bases have been shown to obey the general Brønsted relation in Eq. (5)^44^, where *p* and *q* are statistical factors relating to the number of equivalent H^+^ sites on the acid and the number of basic sites on the base, respectively.

5 $$\log \,{k}_{1}=\beta \left[({\rm{p}}{K}_{{\rm{H}}{\rm{B}}}-{\rm{p}}{K}_{{\rm{H}}{\rm{C}}}),+,\log ,\,,(\frac{{p}_{{\rm{B}}}\,{q}_{{\rm{C}}}}{{q}_{{\rm{B}}}\,{p}_{{\rm{C}}}})\right]+\log \,{k}_{o}+\log \,({q}_{{\rm{B}}}{p}_{{\rm{C}}})$$

Statistical factors were included because they were relatively easy to assign for the small set of carbon acids. For planar enolates, each face of the enolate should be considered a statistically different site of protonation (*q*_C_ = 2) but for this work, we treated planar enolates as having a single basic site (*q*_C_ = 1). Diastereotopic protons were treated as equivalent for XH_2_ acids such as CH_2_. The value of the statistical terms in Eq. (5) correspond to log [(*p*_*B*_*q*_*C*_)*β* • (*q*_*B*_*p*_*C*_)_(1-*β*)_] and ranged from −0.69 to +0.80. Only 2,142 out of 20,859 (10%) of proton transfers from carbon acids involved a statistical term ≥ log(3) = 0.48. Without the inclusion of the statistical factors, 4,258 (20.4%) of the proton transfers from carbon were above the log *k*_1_ = 3.00 cutoff. When the statistical factors were included, 5,043 were above the cutoff; of those, 730 (14%) were raised above the cutoff by a statistical term of log(3) or less. In a typical reaction, the uncertainties in concentrations will have a greater impact on rate = *k*_1_[acid][base] than the statistical factors that affect *k*_1_. These statistical effects are small when considering rate constant ratios on the order of thousands, millions, or billions.

### Assembly of Arrow-Pushing Steps for Proton Transfers Between Carbon and Heteroatoms

Grzybowski and coworkers have trained a system to accurately predict a wide range of equilibrium p*K*_a_s for a wide range carbon acids^45^. Yet, intrinsic rate constants (*k*_o_) and Brønsted β values needed for Eq. (5) are only available for a much smaller number of carbon acids, typically with p*K*_HC_ < 15 and a number of classes of bases such as 1°, 2°, and 3° amines, HO^-^, and carboxylate anions. The carbon acids are typically substituted with one or more anion-stabilizing groups such as nitro, benzoyl, acetyl, carboxyalkyl, cyano, phenyl, pyridinium, phosphonium, and sulfonium. Subsets of bases and p*K*_HB_ values were extracted from the Heteroatom set of 7,613 bases: for example, a set of 1,048 carboxylate anions, 439 primary amines, 380 secondary amines, 857 tertiary amines, 42 thiolate anions, 637 aryloxide anions, and 59 alkoxide anions, including HO^-^ that has the revised p*K*_a_ value of 14.0 for its conjugate acid. For carbon acids, log *k*_o_ and β are dependent on the structure of the base; 81 different pairings were created from 7 heteroatom base classes and 65 specific carbon acids.

**Fig. 4:**
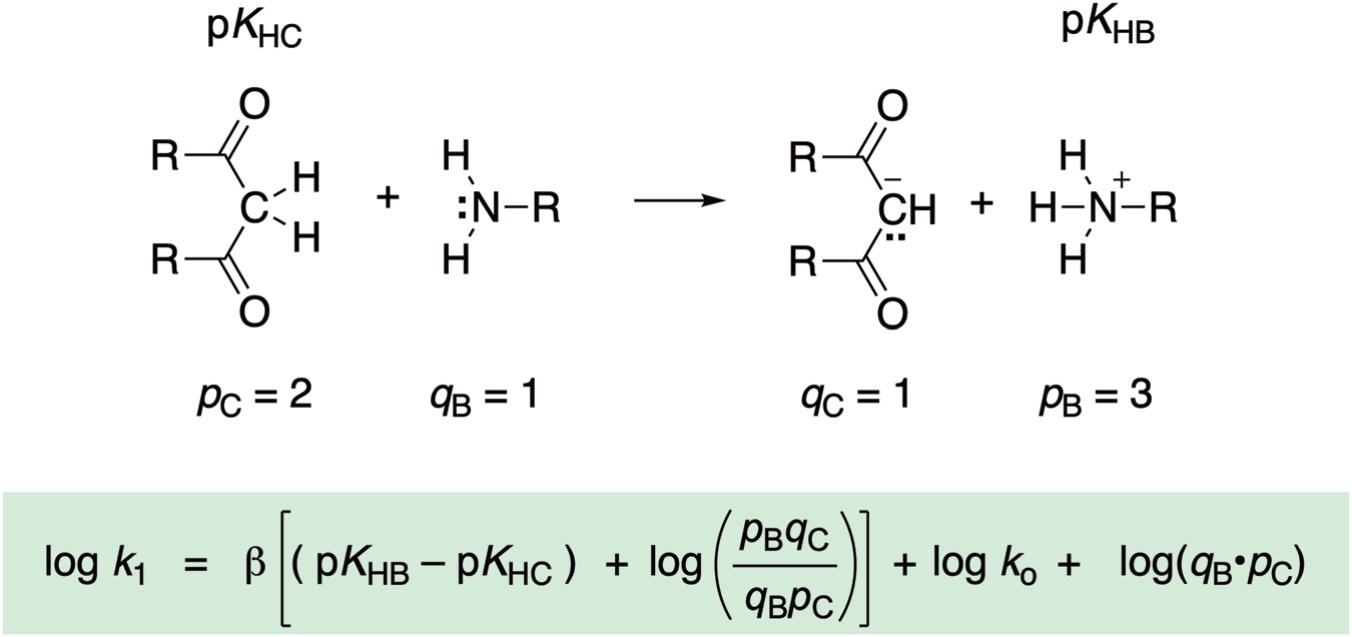
The Eigen-Bernasconi equation relates rate constants for proton transfers (from carbon) to equilibrium p*K*_a_s, intrinsic rate constants, and statistical factors. Log *k*_o_ is dependent on the structure of each carbon acid and each family of heteroatom bases.

We then generated 20,859 combinatorial variations and calculated log *k*_1_ according to the Eigen-Bernasconi equation (Eq. 5, Fig. 4): log *k*_1_ = β [(p*K*_HB_ − p*K*_HC_) + log (*p*_B_ *q*_C_ /*q*_B_ *p*_C_)] + log *k*_o_ + log (*q*_B_ *p*_C_). We included in the dataset proton transfer steps with log *k*_1_ ≥ 3. The resulting set of 5,043 proton transfer steps, from carbon acids to heteroatom bases, were included in the total dataset. Application of this cutoff led to exclusion of proton transfer steps involving 6 out of 65 carbon acids, mostly simple nitro compounds. As a result of the conservative cutoff, there were no remaining examples of tertiary amines deprotonating carbon acids. The final set of proton transfers from carbon acids contained 71 different combinations of the 6 heteroatom base classes with the 59 specific carbon acids.

Experimental determination of rate constants is laborious, and even more so for intrinsic rate constants (*k*_o_) and Brønsted values. Due to the paucity of parameters for proton transfer to carbon bases, the dataset does not contain rate constants calculated for combinatorial variations of carbon bases. To fill this gap, we identified in the literature 49 proton transfers from heteroatom acids to carbon bases (mostly enol ethers and nitronate anions) for which experimentally measured rate constants were available with *k*_1_ ≥ 10^3^ M^−1^ s^−1^ (log *k*_1_ ≥ 3). The advantage of datasets over rules is that additional examples are easily appended.

## Data Records

### Structuring of Data Records

All the datasets are available for download as a single zipped file (10.6084/m9.figshare.30875087)^46^. One folder contains two types of acidity and basicity data that were used to generate millions of raw heteroatom-heteroatom proton transfer steps. They consisted of 7,613 heteroatomic acids (Acid.csv) and 7,613 conjugate bases (ConBase.csv) with structures in SMILES format with p*K*_a_s. Another folder contains eight types of acidity and basicity data (in CSV format) used to generate thousands of raw carbon to heteroatom proton transfer steps. The carbon acids data (Carbon_Acids.csv) consists of names, SMILES, p*K*_a_s, statistical factors *p*_C_ and *q*_C_, intrinsic rate constants (*k*_o_), Brønsted β parameters, and literature references. The data for the seven different classes of bases, each in separate file, consisted of SMILES, p*K*_a_s, statistical factors *p*_B_ and *q*_B_, and literature references (Fig. 4).

These acidity and basicity data were used to generate combinatorial variations of proton transfer steps: i) 51 M proton transfer steps (51M_Heteroatom.csv) from heteroatom acids to heteroatom bases, with SMIRKS, calculated log *k*_1_, and p*K*_a_s, ii) 5 K proton transfer steps from carbon acids to heteroatom bases (5KCarbonPT.csv), with SMIRKS, calculated log *k*_1_, and p*K*_a_s, Brønsted β values, statistical factors (*q*_B_, *p*_B_, *q*_C_, *p*_C_) intrinsic rate constants (*k*_o_), iii) 49 proton transfers from heteroatom acids to carbon bases (49ExperimentalCarbonPT.csv) in SMIRKS format, with experimentally measured log *k*_1_, and literature references. Representative samples of the 51 M heteroatom to heteratom proton transfer steps are also included as CSV files (100K_Heteroatom.csv and 100_Heteroatom.csv).

## Data Overview

**Fig. 5:**
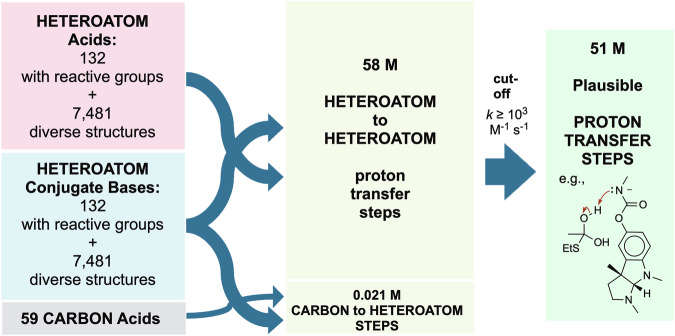
The DataWarrior dataset of structurally diverse heteroatom acids was curated and then complemented with a smaller set of functionally diverse heteroatom acids. Conjugate bases were generated from these acids. Acids and bases were combined to generate 58 M proton transfer steps. A rate factor cutoff was then applied to yield the final set of 51 M Plausible Proton Transfer Steps.

A large dataset was obtained through combinatorial assembly and application of a conservative rate cut-off (Fig. 5). The few examples of carbon bases are important but did not significantly expand the size of the dataset.

**Fig. 6:**
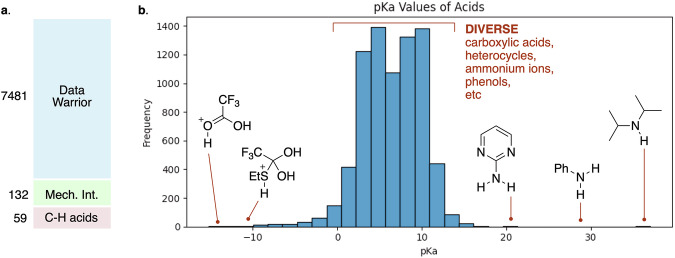
The dataset of proton transfer steps incorporates over 7,600 acidic species with p*K*_a_s spanning a range of over 50 orders of magnitude. (**a**) The proton transfer steps in the dataset involve acids from several sources. (**b**) The set of acids in the proton transfer steps cover a large range of p*K*_a_s, mostly heteroatom acids with experimentally determined p*K*_a_s in the range of 0 to 14.

The resulting dataset of proton transfer steps incorporates over 7,600 acidic species with p*K*_a_s spanning a range from −15 to + 37 (Fig. 6). The majority of the acids come from the DataWarrior dataset, which is a structurally diverse set of heteroatom N, O, and S acid species but covers a relatively limited range of p*K*_a_s. A smaller set of about 100 heteroatom acids was added to ensure that mechanistic intermediates, including species with very high or very low estimated p*K*_a_s, were included. The set of carbon acids is much smaller because the necessary intrinsic rate constants and Brønsted parameters are not widely available. The carbon acids cover a limited range of p*K*_a_s from 5 to 20.

The total dataset of 51,510,157 plausible acid-base proton transfer steps was created combinatorially, using equilibrium aqueous p*K*_a_s to estimate rates of proton transfer. The proton transfer steps are encoded in SMIRKS format with electron-flow specification, which is particularly suitable for machine learning. The majority of the dataset (51,505,065) contains proton transfers to and from heteroatoms; 24,885,144 (48%) of the 51,505,065 entries have log *k*_1_ values calculated by applying p*K*_a_s in the Heteroatom set to Eq. (1). The dataset contains a smaller number (5,043) of proton transfers from carbon acids to groups of heteroatom bases, each with log *k*_1_ from Eq. (5). The dataset also includes a small number (49) of proton transfer steps from heteroatom acids to carbon bases, each with log *k*_1_ values. A conservative cutoff was used to determine plausible steps: log *k*_1_ ≥ 3 for proton transfers between heteroatoms and for proton transfers to or from carbon Table 1.

## Technical Validation

### Validation of Rate Constants - Comparison of Calculated log *k*_1_ with Experimentally Measured log *k*_1_

Numbers in the dataset generated by application of Eq. (1) were compared to published experimental values (Table 2) for proton transfers in protic solvents. The rate constants *k*_1_ in the dataset are generally in good agreement with experimental values, within a factor of 10 M^−1^ s^−1^ (one log unit), for proton transfers spanning *k*_1_ = 10^6^ M^−1^ s^−1^ (log *k*_1_ = 6).

**Table 2:** Comparison of Calculated log *k*_1_ for Heteroatom to Heteroatom Proton Transfer Steps in the Dataset with Reported Experimental Values.

HA (p*K*_HA_)	B (p*K*_HB_)	log *k*_1_ (calc)	log *k*_1_ (exp)	∆ log *k*_1_	Ref
NH_4_^+^ (9.2)	CH_3_CO_2_^–^ (4.7)	4.5	4.32	+0.18	^48^
HCN (9.0)	CH_3_ONH_2_ (4.62)	4.62	5	−0.38	^49^
NH_4_^+^ (9.2)	CH_3_CH_2_CO_2_^–^ (4.83)	4.63	4.3	+0.33	^48^
NH_4_^+^ (9.2)	*t-*BuCO_2_^–^ (5.04)	4.84	4.58	+0.26	^48^
PrNH_3_^+^ (10.65)	imidazole (7.07)	5.42	6.15	−0.73	^30^
CH_3_CO_2_H (4.7)	Cl_2_CHCO_2_^–^ (1.48)	5.78	6.76	−0.98	^30^
CH_3_CO_2_H (4.7)	ClCH_2_CO_2_^–^ (2.85)	7.15	7.38	−0.23	^30^
imidazoleH^+^ (7.07)	pyridine (5.26)	7.19	7.43	−0.24	^30^
imidazoleH^+^ (7.07)	ɑ-picoline (5.98)	7.91	7.81	+0.10	^30^
malonic acid (5.7)	CH_3_CO_2_^– ^(4.7)	8.0	7.79	+0.21	^30^

Numbers in the dataset that were generated by application of Eq. (5) to proton transfers from carbon acids were compared to published experimental values (Table 3) in aqueous solvents or aqueous solvent mixtures from 20-25 °C. As with transfers between heteroatoms, most of the calculated log *k*_1_ values were within an order of magnitude of the experimental values.

**Table 3:** Comparison of Calculated log *k*_1_ for Carbon to Heteroatom Proton Transfer Steps in the Dataset with Reported Experimental Values.

HA (p*K*_HA_)	B (p*K*_HB_)	log *k*_1_ (calc)	log *k*_1_ (exp)	∆ log *k*_1_	Ref
PhSCH_2_NO_2_ (6.67)	piperazine (9.93)	3.16	3.02	+0.14	^50^
PhCOCH(Ph)NO_2_ (5.04)	MeO_2_CCH_2_CH_2_S^–^ (9.33)	3.25	3.02	+0.23	^51^
PhCOCH_2_NO_2_ (4.67)	PhO^–^ (9.9)	3.54	3.51	+0.03	^51^
PhSO_2_CH_2_(4-Py^+^)Me (11.54)	*n*-BuNH_2_ (10.7)	3.90	3.57	+0.33	^44^
indanedione (6.35)	*n*-BuNH_2_ (10.7)	4.64	4.16	+0.48	^52^
AcCH_2_PPh_3_^+^ (7.83)	MeOCH_2_CH_2_NH_2_ (9.67)	5.02	5.16	−0.14	^44^
indanedione (6.35)	piperidine (11.24)	5.39	4.89	+0.50	^52^

Values for log *k*_1_ are reported to the hundredths decimal place (like most of the literature p*K*_a_ values), but the calculated log *k*_1_ clearly lacks this high level of precision. For users training a system to distinguish plausible proton transfer from implausible proton transfers, the levels of accuracy afforded by the Eigen relationship are sufficient.

## Data Availability

All the datasets are available for download as a single zipped file in the PMechDB section at DeepRXN^47^ (https://deeprxn.ics.uci.edu/pmechdb/download) and at figshare (10.6084/m9.figshare.30875087) under a CC-BY license^46^.

## Code availability

The codes used to generate the proton transfer steps are publicly available at (https://github.com/rjmille3/combinatorial-proton-transfer.git).
